# The development of executive function and language skills in the early school years

**DOI:** 10.1111/jcpp.12458

**Published:** 2015-08-26

**Authors:** Debbie Gooch, Paul Thompson, Hannah M. Nash, Margaret J. Snowling, Charles Hulme

**Affiliations:** ^1^ Department of PsychologyRoyal HollowaySurreyUK; ^2^ Department of Experimental PsychologyUniversity of OxfordOxfordUK; ^3^ Department of PsychologyUniversity of LeedsLeedsUK; ^4^ Division of Psychology and Language SciencesUniversity College LondonLondonUK

**Keywords:** Executive function, language skills, family risk of dyslexia, language impairment, longitudinal, development

## Abstract

**Background:**

The developmental relationships between executive functions (EF) and early language skills are unclear. This study explores the longitudinal relationships between children's early EF and language skills in a sample of children with a wide range of language abilities including children at risk of dyslexia. In addition, we investigated whether these skills independently predict children's attention/behaviour skills.

**Method:**

Data are presented from 243 children at four time points. Children were selected for being at risk of reading difficulties either because of a family history of dyslexia (FR;* N* = 90) or because of concerns regarding their language development (LI;* N* = 79) or as typically developing controls (TD;* N* = 74). The children completed tasks to assess their executive function and language skills at ages 4, 5 and 6 years. At 6 (T4) and 7 years (T5) parents and teachers rated the children's attention/behaviour skills.

**Results:**

There was a strong concurrent relationship between language and EF at each assessment. Longitudinal analyses indicated a considerable degree of stability in children's language and EF skills: the influence of language on later EF skills (and vice versa) was weak and not significant in the current sample. Children's EF, but not language, skills at T3 predicted attention/behaviour ratings at T4/T5.

**Conclusions:**

There is a strong concurrent association between language and EF skills during the preschool and early school years, when children with language impairment show persistent EF deficits. Latent variables measuring language and EF show high longitudinal stability with little evidence of significant or strong reciprocal influences between these constructs. EF, but not language, skills predict later ratings of children's attention and behaviour.

## Introduction

Executive function is a multidimensional construct involving skills such as attention control, behavioural inhibition and working memory, each important for the deliberate control of goal orientated actions (Welsh, Pennington, & Groisser, 1991; Zelazo & Müller, 2002). It follows that executive function can be considered critical for ‘school readiness’ (Bierman, Nix, Greenberg, Blair, & Domitrovich, 2008; Shaul & Schwartz, 2014) and classroom learning (Blair & Razza, 2007; see Liew, 2012 for a review). In addition, although some measures of executive function are more closely related than others, all share common variance (Miyake et al., 2000) and a relatively stable common executive function factor is predictive of individual differences in externalising behaviour problems (Miyake & Friedman, 2012).

Weaknesses in executive function have been reported not only in many neurodevelopmental disorders, notably ADHD (Barkley, 1997) but also autism spectrum disorders (Pennington & Ozonoff, 1996) and several lines of evidence suggest that executive functions are closely related to language. First, correlations between executive function and language skills are frequently reported (e.g. Carlson, Davis, & Leach, 2005; Gooch, Hulme, Nash, & Snowling, 2013; Müller, Jacques, Brocki, & Zelazo, 2009) leading to the hypothesis that children's use of language may facilitate their performance on executive function tasks (Brace, Morton, & Munakata, 2006; Kirkham, Cruess, & Diamond, 2003; Zelazo, Reznick, & Pinon, 1995). Consistent with this view, executive function deficits have been reported in children with language impairment (Gooch et al., 2013; Henry, Messer, & Nash, 2012; Wittke, Spaulding, & Schechtman, 2013). More generally, Kuhn, Willoughby, Wilbourn, Vernon‐Feagans, and Blair (2014) found that early language skills had both direct and indirect effects on children's later executive functions; relatedly, language ability predicts later behaviour problems in children (Lindsay, Dockrell, & Strand, 2007; Petersen et al., 2013). This study focuses on the developmental relationships between executive function and language skills during the preschool and early school years and considers whether there is evidence of a causal relationship between these skills and ratings of children's behaviour and attention.

Bishop, Nation, and Patterson (2014) suggest three possible models of the relationship between weaknesses in executive function and language impairment; (a) executive functions exert a causal influence on the development of language (e.g. good attentional skills may facilitate language learning); (b) language skills are causally related to the development of executive function, perhaps because children use verbal mediation to help them perform some executive function tasks; in line with this is the proposal of Barkley (1997) that ‘inner speech’ (or verbal working memory) is a core component of executive function; (c) there is no direct causal relationship at the cognitive level between language and executive function skills but it is plausible that shared genetic risk factors affecting neuronal migration and the consequent development of brain systems could account for the correlations between these skills observed during early development. However, the causal relationships described in (a) and (b) above are not mutually exclusive and a further alternative is that executive functions and language skills may develop in reciprocal interaction and the effects of one on the other could change over time. Longitudinal studies such as the one reported here provide a useful starting point for assessing the plausibility of these different causal relationships.

Until recently, there has been little research assessing the direction of possible causal relationships between executive function and language skills in children. Kuhn et al. (2014) explored the relationship between children's early communicative gestures (measured at 15 months), language skills (measured at 2 and 3 years) and later executive function skills (measured at 4 years) in a large epidemiological sample. They showed that children's early language skills predicted their later executive function skills. However, the autoregressive effect (i.e. earlier measures of executive function) was unaccounted for in their analyses and it therefore remains unclear whether children's language skills predict their executive function skills once pre‐existing levels of executive function are controlled.

Petersen, Bates, and Staples (2014) explored the role of language ability and self‐regulation in the development of inattentive–hyperactive behaviour problems during the preschool years. Like Kuhn et al. (2014), they found that language ability predicted later self‐regulation which, in turn, mediated the effect of language on later inattentive–hyperactive behaviour ratings. However, the effect of self‐regulation on later language was not significant over and above the effect of pre‐existing language skills. Moreover, given the large amount of missing data in this study, this finding is in need of replication.

In summary, there is a need for longitudinal evidence investigating the relationships between executive function, language skills and later measures of attention and behaviour. Since both executive and language skills are crucial foundations for learning, we chose to examine the relationships between these skills from preschool to the start of formal schooling. Our sample were children selected to be at high risk of a specific learning disorder either because of a family history of dyslexia or because of concerns regarding their speech and language development at age 3 ½ years and age‐matched controls. Our primary research question was to clarify how language skills and executive functions relate to each other in early childhood. Specifically, if language difficulties cause deficits in executive function we predicted that children's early language skills would predict their later executive function skills. Conversely, if deficits in executive function cause language difficulties we predicted that children's early executive function skills would predict their later language skills. In order to test these alternative models we followed children over 3 years from ages 4–5 years (preschool) through the first 2 years of formal education (ages 5–6 years and 6–7 years) assessing their language and executive skills at each time point. In addition, we aimed to replicate findings suggesting that language skills and executive functions each predict ratings of children's behavioural adjustment (e.g. Beitchman et al., 1996).

## Method

Data are reported from four phases (T2‐T5) of a prospective longitudinal study of children at high risk of dyslexia. After joining the study at approximately 3.5 years old (T1) children were assessed at approximately annual intervals; once again in the preschool period (aged 4–5 years, T2) and twice after they started school (aged 5–6 years, T3 and 6–7 years, T4); ratings of children's behaviour and attention were obtained at T4 and T5 (7–9 years). Data from T1 are reported elsewhere (Nash, Hulme, Gooch, & Snowling, 2013).

Ethical approval for the study was granted by the NHS Research Ethics Committee and the University of York, Department of Psychology Research Ethics Committee. Parents provided informed consent for their child to participate.

### Participants

Children and their families were recruited to the Wellcome Language and Reading project for being at risk of reading difficulties either because of a family history of dyslexia (FR; *N* = 90) or because of concerns regarding their language development (Language concerns; *N* = 79) or as controls (TD; *N* = 74). Recruitment was via advertisements and webpages, and via speech and language therapy services in Yorkshire, UK (full details of ascertainment procedures and ‘diagnostic groupings’ are given in Nash et al., 2013). Of the total 243 children, 141 (58%) were male and none met our exclusionary criteria at T1/T2 (MZ twinning, chronic illness, deafness, English as an additional language, care provision by local authority and known neurological disorder, for example cerebral palsy, epilepsy, ASD). Although in the main project, at‐risk children were classified as being at family risk of dyslexia (FR) and/or as having specific language impairment (SLI) resulting in four groups, for the main analyses reported here, data were treated as one group comprising a wide range of language abilities. We also ran parallel sets of analyses for the at‐risk children (FR and SL‐concerns) and the controls (TD). There was a small amount of attrition between time points (T2‐T3 *N* = 2, T3‐T4 *N* = 2, T4‐T5 *N* = 5).

Given our recruitment procedure, the sample was weighted towards those with language learning difficulties and hence the language scores span a wide range of ability. Table 1 shows descriptive statistics for the sample including background variables, measures of language and executive function taken at T2‐T5 and ratings of children's behaviour and attention (SWAN) taken at T4/T5.

**Table 1:** Sample descriptives for measures taken at T2‐T5

Variables	*N*	Mean (*SD*)	Min	Max	Skew
Age at T2 (months)	243	56.23 (3.63)	50	67	.83
Age at T3 (months)	240	67.98 (3.41)	60	78	.24
Age at T4 (months)	240	78.58 (4.39)	67	90	.27
SES (based on postcode; %)^a^	243	63.30 (28.07)	3	99	.16
IQ score T2^b^	241	106.57 (19.07)	58	148	.15
T2 Language measures					
ROWPVT	242	60.50 (10.57)	26	89	−.19
CELF sentence structure	243	16.61 (3.26)	2	22	−1.32
Experimental Sentence Imitation Task (ESIT)	220	5.65 (3.98)	0	19	.62
T2 Executive function measures					
Block recall	234	15.62 (3.99)	1	24	−.10
Visual search efficiency	237	.16 (.07)	.08	.30	−.77
HTKS	231	20.42 (12.27)	0	39	−.33
T3 Language measures					
CELF expressive vocabulary	240	26.99 (8.91)	2	47	−.29
CELF sentence structure	240	20.32 (3.86)	8	26	−.86
ESIT	236	8.16 (4.67)	0	19	.28
T3 Executive function measures					
Block recall	240	19.05 (3.82)	9	30	−.07
Visual search efficiency	237	.20 (.05)	.05	.44	−1.24
HTKS	237	27.79 (10.16)	0	40	−1.20
T4 Language measures					
CELF expressive vocabulary	240	33.29 (9.27)	2	52	−.82
ROWPVT	240	81.07 (13.34)	40	130	.29
ESIT	239	9.89 (4.80)	0	20	−.13
T4 Executive function measures					
Block recall	240	20.42 (4.19)	6	30	−.38
Visual search efficiency	239	.26 (.08)	.02	1	−.65
GoNoGo	236	5.11 (3.38)	0	17	.97
SWAN ratings					
T4 teacher	175	78.99 (21.10)	29	125	−.03
T5 teacher	135	85.10 (23.11)	22	126	−.28
T4 parent	174	78.71 (16.59)	0	126	−.55
T5 parent	155	78.85 (17.01)	19	119	−.11

a SES based on postcode in United Kingdom, relative rank according to deprivation value; Lower = more deprived (Department of Communities and Local Government, Indices of Multiple Deprivation 2007).

b NVIQ is standard score (T2 WPPSI‐III Block Design).

### Tests and procedures

Children were administered numerous cognitive, language and literacy tests at each time point. Here, we report only those measures used in the present analyses. At T2 assessments were conducted at home during two 1‐hour sessions with breaks as necessary. At T3 and T4 assessments were conducted at school during a 2‐hr session with breaks.

#### Language measures

*Receptive vocabulary (Receptive One Word Picture Vocabulary Test (ROWPVT) Brownell,* 2000) (T2, T4). The child heard a word and was asked to select the corresponding picture, from a choice of four (*α* = .95).

*Expressive vocabulary (CELF‐4 UK; Semel & Wigg,* 2006 *(T3, T4))*. The child was asked to name objects (e.g. carrot, telescope) or to describe what a person is doing (e.g. riding a bike) (*α *= .78–.82).

*Sentence structure (CELF‐Preschool 2 UK;* Semel, Wigg, & Secord, 2006 *(T2) & CELF‐4 UK; Semel & Wigg,* 2006 *(T3))*. The child heard a sentence (e.g. the bear is in the wagon) and had to select from a choice of four, the picture that conveyed its meaning. The sentences included a range of different syntactic structures (*α* = .78–.83).

*Experimental Sentence Imitation Task (ESIT) (T2, T3, T4)*. The child was asked to repeat 20 sentences varying in length (short vs. long) and complexity (transitive vs. ditransitive) (e.g. ‘a lady pushed the bike to work’ and ‘the busy teacher promised the clever boy a sticker’). The total number of sentences repeated correctly was recorded and used in the current analyses (*α* = .78).

#### Executive function measures

*Head Toes Knees and Shoulders (HTKS) task* (Burrage et al., 2008) *(T2, T3)*. In this measure of behavioural inhibition the child had to do the opposite of what the examiner said (e.g. touch their toes if asked to touch their head and vice versa). If the child was able to successfully inhibit on 5/10 trials they went on to complete a further block of 10 harder trials with additional commands (e.g. to touch their shoulders if asked to touch their knees and vice versa). Each correct response received two points, self‐corrected responses (partial inhibitions; where the child moved towards the incorrect, intuitive response but demonstrated the correct final response) received 1 point and incorrect responses received 0 points (max score = 40). Stability between T2 and T3; *r *=* *.52.

*Visual Search* (the *Apples Task*; Breckenridge, 2008) *(T2, T3, T4)*. The child was given 1 min to search an array to identify targets (18 red apples) whilst ignoring distracters (81 red strawberries and 81 white apples). The number of targets identified and the number of commission errors made (pointing to a distracter; false alarms) were recorded. A visual search efficiency score ((Hits: total targets correctly identified – commission errors)/60 s) was calculated and used in the current analyses; a high score reflects better selective attention. Stability between T2‐T3 and T3‐T4; *r *=* *.59 and .49 respectively.

*Block Recall (Working Memory Test Battery for Children,* Pickering & Gathercole, 2001 *) (T2, T3, T4*). This task was used to measure visuo‐spatial memory. The child saw the examiner tap a sequence of blocks on a board and then recalled the sequence by tapping the blocks in the same order. The number of correct trials was recorded (max 52). Test–retest reliability = .63.

*Go/No‐Go (T4)*. In this computerised behavioural inhibition task children completed 80 Go/NoGo trials; on 75% of these trials the go stimulus was presented (bug) and on 25% of the trials the no‐go stimulus was presented (ladybird). Prior to the GoNoGo trails children complete 30 go trials to establish the prepotent/automatic response, which they then had to try and inhibit on the no‐go trials. Children were instructed to press a button as quickly as possible (within 2000 ms) when they saw the bug but not when they saw the ladybird. The task lasted for approximately 5 min and the number of commission errors made on no‐go trials was used as an index of behavioural inhibition.

#### Ratings of children's behaviour and attention

*The Strengths and Weaknesses of ADHD symptoms and Normal‐behaviour Questionnaire* (*SWAN*; Swanson et al., 2006 *)* was completed by parents and teachers at T4, and T5*)*. This scale captures strengths as well as weaknesses in attention/behaviour skills (Polderman et al., 2007).

The items on the SWAN map onto the symptoms of ADHD and include nine items tapping Inattention and nine items tapping Hyperactivity/Impulsivity. Here, we focus on a composite measure of attention and behaviour since the objective was to measure outcomes continuously rather than for diagnosis. For each item respondents were asked to compare their child's attention/behavioural skills to those of his/her peers using a seven point Likert scale (Far Below Average = 1, Below Average = 2, Somewhat Below Average = 3, Average = 4, Somewhat Above Average = 5, Above Average = 6, and Far Above Average = 7). The maximum score on the SWAN is 126. A low score reflects weaknesses in attention/behavioural skills.

## Results

Analyses were conducted using Mplus 6.1 (Muthén & Muthén, 1998‐2011) with missing data being handled using Full Information Maximum Likelihood estimation.

Multiple measures of language and executive function were administered so we performed confirmatory factor analyses (CFA) on the measures at each time point (see Figure S1, available online). These models show that, at each time point, separate but correlated factors for executive functions (EF) and language fitted the data well confirming that language and EF are partially separable constructs. At each time point, constraining the correlation between the EF and language factors to 1.0 (which is equivalent to specifying EF and language as a single latent construct) resulted in a significant loss of fit confirming that EF and language are best conceptualised as separate though correlated constructs (T2 Δχ^2^ (*df* 1) = 13.83, *p *=* *.0002; T3 Δ χ^2^ (*df* 1) = 19.473, *p *=* *.00001; T4 Δ χ^2^ (*df* 1) = 9.366, *p* = .002).

**Figure 1:**
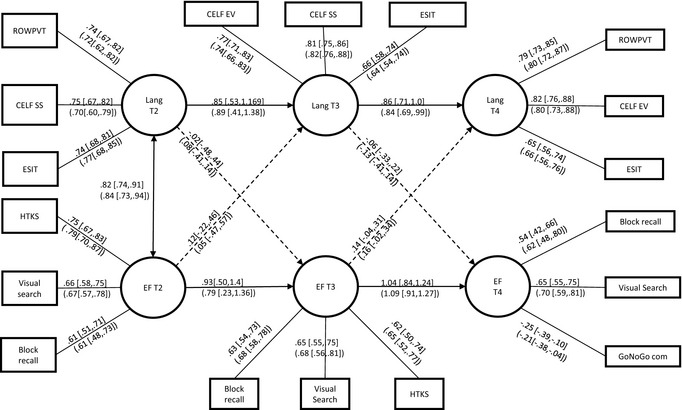
10Path diagram showing the relationship between executive function and language ability in children aged 4–7. Path weights and confidence intervals for the total sample are shown outside the brackets, those for the at‐risk only sample are shown inside brackets. Note: Two multivariate (T3 executive functions (EF) vs. T4 Lang) outliers were excluded (1 LI and 1 FR)

To assess how language and executive skills relate to each other longitudinally, a latent variable autoregressive path model with cross‐lagged effects was fitted to the data for the whole sample, and separately for the at‐risk sample only (children with speech‐language concerns, family‐risk status or both). This model (see Figure 1) assesses the longitudinal stability of language skills (receptive and expressive vocabulary, sentence structure, ESIT) and executive function (block recall, visual search, HTKS, GoNoGo) and also assesses whether either of these constructs predicts additional variance in the other construct across successive time points. If such longitudinal cross‐loadings were present they would be consistent with (but not prove) a causal influence from the earlier to the later variable.

We assessed the cross‐lagged effects by fitting four models, where each cross‐lagged effect was removed systematically and the chi‐square difference calculated. (a) The first model included all cross‐lagged paths; (b) the path between executive function at T2 and Language at T3 was dropped; (c) the path between language at T2 and executive function at T3 was dropped; (d) the path between language at T4 and executive function at T3 was dropped; (e) the path between executive function at T4 and Language at T3 was dropped; (f) Finally, all cross‐lagged paths were removed. The last of these models, shown in Figure 1, is the most parsimonious and gives a good fit to the data for the sample as a whole (χ^2^ (116) = 140.654, *p *=* *.06, CFI = .988, RMSEA = .030 (90% CI .000–.046), SRMR = .039) and for the at‐risk only group (χ^2^ (117) = 146.007, *p* =* *.04, CFI = .980, RMSEA = .038 (90% CI .011–.057), SRMR = .046). The figure shows standardised path weights together with their 95% confidence intervals (coefficients for whole sample outside the brackets; those for the at‐risk sample only inside the brackets; paths significant at the 0.05 level are represented by solid lines; nonsignificant paths by dashed lines).

A number of features of this model are noteworthy. First, the latent variables describing language and executive function show very high stability despite minor differences in the measures used to define them across time points. The model accounted for 88% and 94% of the variance in language skills at T3 and T4, respectively, and 85% and 100% of the variance in executive function at T3 and T4 respectively. Second, all cross‐lagged effects are small and none are close to being significant (with the possible exception of the path from T3 executive function → t4 language). Finally, the coefficients for the at‐risk children show an essentially identical pattern to that for the full sample.

**Figure 2:**
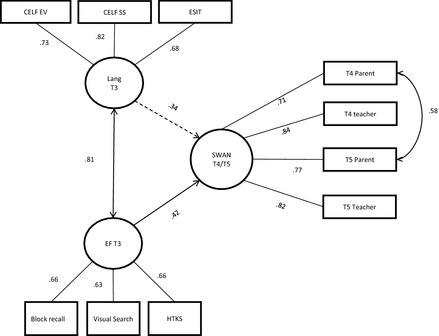
Path diagram showing language and executive function as predictors of ADHD symptoms (SWAN)

The model in Figure 2 assesses the relationship between language and executive function at T3 and ratings of children's behaviour and attention at T4/T5. The model provides a good fit to the data (χ^2^ (31) = 40.831, *p *=* *.111, CFI = .987, RMSEA = .036 (90% CI .000–.064), SRMR = .087). Although there is a high correlation between the executive function and language latent variables (*r *=* *.81), it is notable that the path from executive function to attention/behaviour (SWAN) was significant (standardised effect = .42; *p *=* *.033) while that from language was not (standardised effect = .34; *p *=* *.074). In short, ratings of children's behaviour and attention are predicted by executive function independently of language (which does not make any additional significant contribution once the effects of executive function are controlled).

## Discussion

This study explored the relationship between language skills and executive function in children selected to be at‐risk of language learning impairments (due to a family history of dyslexia or concerns about their language development in preschool), during the transition from preschool to formal schooling. Longitudinal path models show that there is very strong stability in both children's language skills and executive function from preschool into the early school years. Such strong stability makes finding significant cross‐lagged effects difficult, and there was little evidence of any such effects over the time period studied here (cross‐lagged path weights were small and not significant). In line with the findings of Petersen et al. (2014), executive functions predicted later ratings of children's attention and behaviour but language skills did not.

Our findings help to clarify the relationships between the development of language skills and executive function. First, the cross‐lagged effects from language skills to executive function were weak, indicating that it is unlikely that language difficulties cause deficits in executive function. The cross‐lagged effects from executive function to language skills were slightly stronger, but still not significant, which indicates that it is unlikely that executive functions provide strong constraints on language development. The only cross‐lagged effect that approached being significant was that from EF at T3 to language at T4. With a larger sample an effect of this magnitude would be significant; nevertheless the current data suggest effects are weak at best. The absence of any substantial cross‐lagged effects (singular or reciprocal) questions the view that improvements in one domain will have knock‐on effects on the other at least across the age range studied here (4–7 years). However, these findings do not rule out the possibility that early language skills could promote the development of executive skills before age 4, or vice versa. Indeed, it is hard to refute the view that attentional capacities in infancy are crucial for the development of language (e.g. Baldwin, 1995) or similarly those young children with better developed language will be more likely to deploy these in order to understand and to complete executive function tasks.

The strong concurrent relationship between language and executive skills found at the first time point raises the possibility that a third factor not measured here could account for their relationship. For example, a general factor such as processing speed could account for the development of both executive and language skills (e.g. Im‐Bolter, Johnson, & Pascual‐Leone, 2006; Leonard et al., 2007). Relatedly, if shared genetic mechanisms are involved in the development of both language and of executive skills, it is plausible that delayed development of the frontal lobes may impinge on brain regions important for executive function, and on adjacent areas implicated in language processing (Bishop et al., 2014). Such genetic effects could cause correlated but distinct patterns of development as found here. One consequence of such shared genetic effects would be the comorbidity, which is so frequently observed between disorders of executive and language function in a variety of neurodevelopmental disorders.

To gain better evidence for possible causal influences of language skills on executive function (or vice versa) training studies are needed. Early interventions to improve oral language skills in children with language weaknesses have been demonstrated to be effective (e.g. Bowyer‐Crane et al., 2008; Fricke, Bowyer‐Crane, Haley, Hulme, & Snowling, 2013), but as yet there is no evidence of their effects on executive skills. Likewise, although a number of diverse activities and curricula have been shown to improve children's executive functions (Diamond & Lee, 2011), evidence that their benefits extend beyond executive control to language or reading is lacking. Indeed there is no evidence that interventions which are focused on improving specific aspects of executive function (e.g. working memory training programmes) show far transfer to language or reading skills (e.g. Melby‐Lervag & Hulme, 2013 for a review).

This study had a number of limitations including the use of different measures at the three time points, which was necessitated by the need to measures children's abilities sensitively at different ages. However, the study is unique in having followed a relatively large sample of children with a wide range of language skills over a critical developmental period. Our findings suggest that executive function and language skills have separate but correlated origins and neither skill strongly predicts the other longitudinally, once autoregressive effects are controlled. It follows that, although executive deficits are commonly seen in children with language impairment, each appears to have a distinct developmental course and deficits in each may require different interventions.

**Key points**

- The nature of the developmental relationship between language and executive function to date is unclear.
- There is a strong concurrent relationship between language and executive function.
- There is considerable longitudinal stability of both language and executive function over the preschool and early school years.
- Children's executive function skills, but not their language skills, are longitudinal predictors of behaviour and attention.

## Supporting information


**Appendix S1.** Detailed task descriptions.
**Figure S1**. Research criteria for LI.
**Figure S2.** Confirmatory factor analysis of the language and executive function variables.
