# Supplementary material for: The development of executive function and language skills in the early school years
Source: J Child Psychol Psychiatry. 2015 Aug 26;57(2):180–7. doi: 10.1111/jcpp.12458 (PMC4832357; doi:10.1111/jcpp.12458)
Supplement: Supplementary file 1 — Appendix S1. Detailed task descriptions. Figure S1. Research criteria for LI. Figure S2. Confirmatory factor analysis of the language and executive function variables. [file JCPP-57-180-s001.docx]

**Supporting information for *The development of executive function and language skills in the early school years* by Gooch et al.**

**Figure S1**


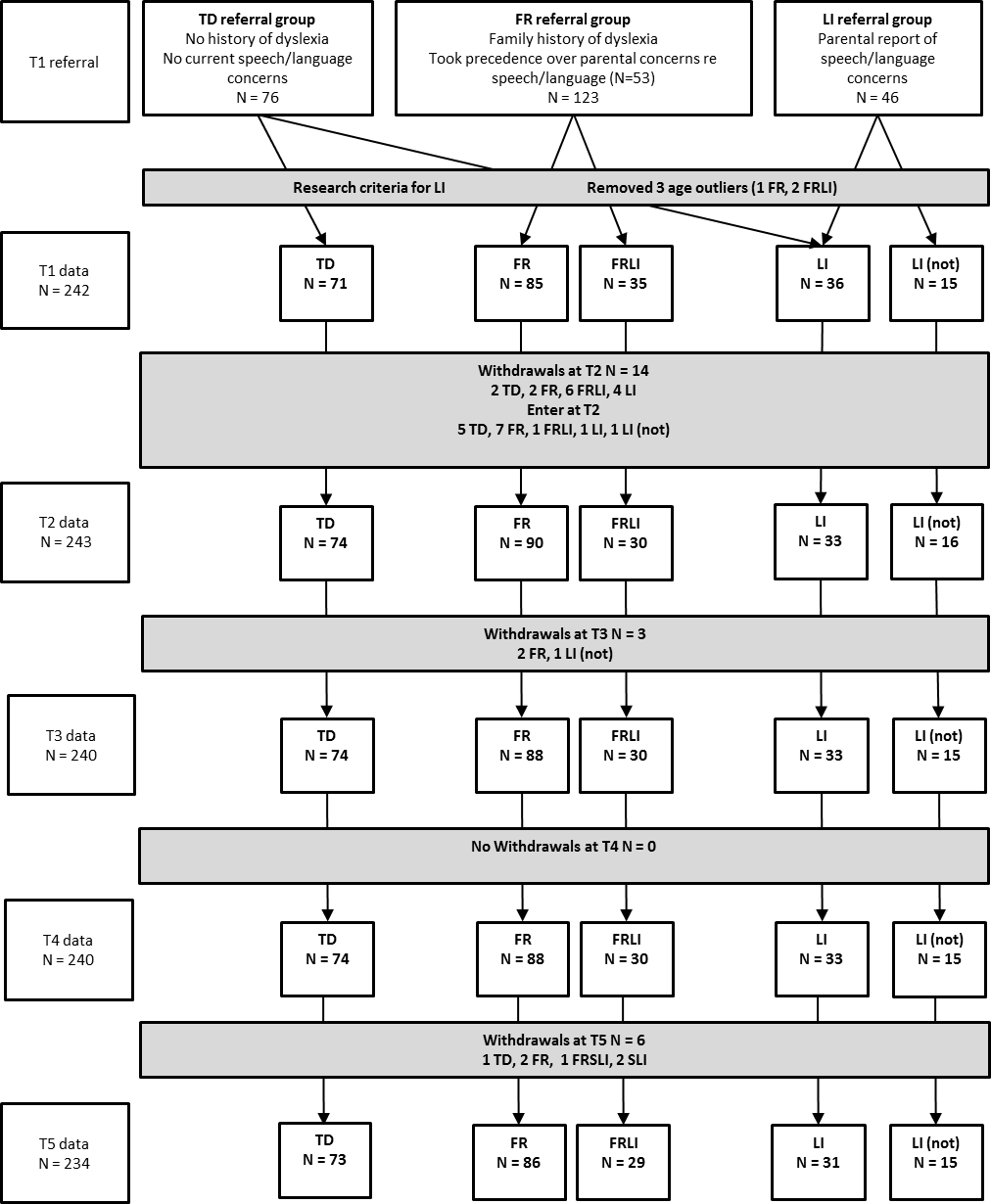


Note:

Research criteria for LI at T1: Failure of 2/4 language tests given at T1 (e.g. A standard score of 85 or less on subtests from the CELF-Preschool 2 (Semel & Wiig, 2006); basic concepts, expressive vocabulary and sentence structure and/or failed the screener from the TEGI (Rice & Wexler, 2001); but passed phonological probe). Where insufficient data, PSep, TEGI subtests, T2 CELF sentence structure and clinical judgement were used. Research criteria for LI at T2: Failure of 2/4 language tests given at T2 (e.g. A standard score of 85 or less on CELF sentence structure, Receptive One Word Picture Vocabulary Test, Preschool Repetition Test and/or failed the TEGI).

**Appendix S1: Detailed task descriptions**

*Executive function measures*

*Head Toes Knees and Shoulders (HTKS) task* (Burrage et al., 2008) *(T2, T3).* In this measure of behavioural inhibition the child was first asked to touch their head and then their toes. Once it had been established that they could perform this action they were instructed to do the opposite of what the examiner said (e.g. touch their toes if asked to touch their head and vice versa). The child had 4 opportunities to practice this, with up to 3 re-explanations of the directions. They then completed a block of 10 test trials with no feedback. If the child was able to successfully inhibit on 5/10 trials they went on to complete a further block of harder trials. For these trials, additional commands to touch their shoulders and knees were added and the child was reminded to do the opposite of what the examiner said (e.g. touch their shoulders if asked to touch their knees and vice versa). After four practice trials the child completed 10 further test trials involving all four commands in a predetermined, pseudorandom order. Each correct response received 2 points, self-corrected responses (partial inhibitions; where the child moved towards the incorrect, intuitive response but demonstrated the correct final response) received 1 point and incorrect responses received 0 points (max score = 40). Stability between T2 and T3; *r* = .52.

*Visual Search (T2, T3, T4).* A visual search task (the *Apples Task*; Breckenridge, 2008), which comprised an array of targets (18 red apples) and distracters (81 red strawberries and 81 white apples), was used to assess selective attention. The child was given 1 minute to search the array and point to targets whilst ignoring distracters. The number of targets identified and the number of commission errors made (pointing to a distracter; false alarms) were recorded. A visual search efficiency score ((Hits: total targets correctly identified – commission errors)/60 seconds) was calculated and used in the current analyses; a high score reflects better selective attention. Stability between T2-T3 and T3-T4; *r* = .59 and .49 respectively.

*Block Recall (Working Memory Test Battery for Children, Pickering & Gathercole, 2001) (T2, T3, T4*). This task was used to measure visuo-spatial memory. The child saw the examiner tap a sequence of blocks on a board and then recalled the sequence by tapping the blocks in the same order. The number of correct trials was recorded (max 52). Test-retest reliability = 0.63.

*Go/No-Go (T4).* This computerised task measures both simple reaction time and behavioural inhibition (the ability to withhold an ongoing response). First children completed 30 simple reaction time trials preceded by 3 practice trials to establish a prepotent or automatic response. On each trial a fixation cross was presented (for either 300, 600 or 900ms) followed by the stimulus (beetle picture for 500ms) after which the screen went blank. Children were instructed to press the middle button on the button box as quickly as possible when they saw the bug. Children had 2000ms from the onset of the stimulus to make their response. When the response button was pressed a blue splat appeared on the screen for 500ms if the button was not pressed ‘too slow…’ appeared for 500ms. Immediately following the reaction time trials the Go/No-Go procedure was explained and children were presented with 8 practice Go/No-Go trials. First a fixation cross was presented (for either 300, 600 or 900ms). The stimulus was then presented for 500ms after which the screen went blank. On 75% of the trials the go stimulus was presented (bug) and on 25% of the trials the no-go stimulus was presented (ladybird). Children were instructed to press the button as quickly as possible when they saw the bug but not when they saw the ladybird. Children had 2000ms from the onset of the stimulus to make their response. When the button was pressed a blue splat appeared on the screen for the remainder of the time (i.e. 2000 – Target2.RT). If the button was not pressed the screen remained blank for 1500ms. The average ISI was 2600ms (range 2300-2900). Trials were presented in a pseudo-random order. Following the practice trials children completed 80 Go/No-Go trials. The task lasted for approximately 5 minutes and the number of commission errors made on No-Go trials was used as an index of behavioural inhibition.

*Behavioural outcomes*

*The Strengths and Weaknesses of ADHD-symptoms and Normal-behaviour Questionnaire* (*SWAN*; Swanson et al., *2006)* was completed by parents and teachers at T4, and T5*).* This scale captures strengths as well as weaknesses in attention/behavior skills (Polderman et al., 2007).

The items on the SWAN, which are positively worded statements, map onto the 18 DSM-IV symptoms of ADHD and include 9 items tapping Inattention and 9 items tapping Hyperactivity/ Impulsivity. For each item respondents were asked to compare their child’s attention/ behavioural skills to those of his/her peers using a 7 point Likert scale (Far Below Average = 1, Below Average = 2, Somewhat Below Average = 3, Average = 4, Somewhat Above Average = 5, Above Average = 6, and Far Above Average = 7). The maximum score on the SWAN is 126 (63 for each of the subscale: Inattention and Hyperactivity/Impulsivity). A low score reflects weaknesses in attention/behavioural skills.

(a)


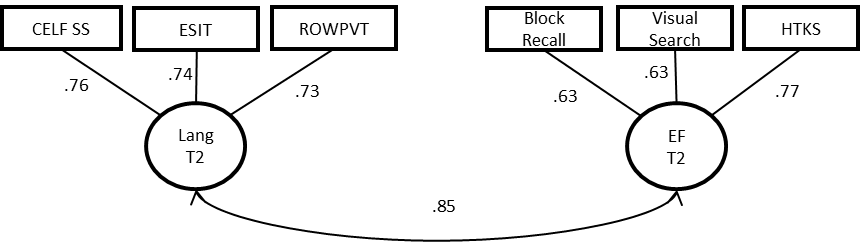


$\chi^{2}$(8) = 7.588, p= 0.475; RMSEA = 0.000; CFI = 1.000.

(b)


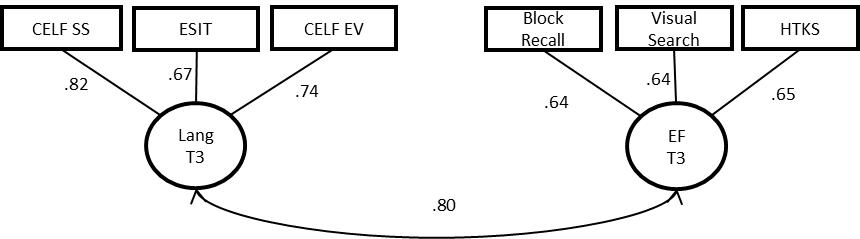


$\chi^{2}$ (8) = 9.833, p= 0.277; RMSEA = 0.031; CFI = 0.995.

(c)


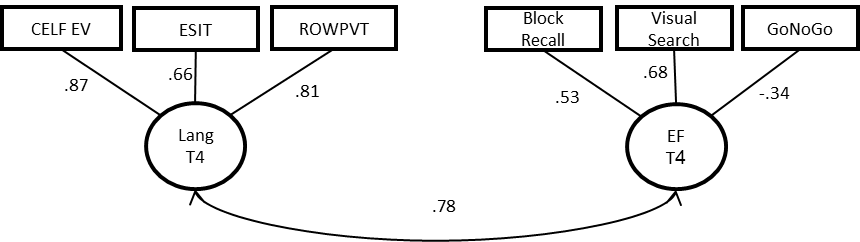


$\chi^{2}$ (8) = 10.941, p= 0.205; RMSEA = 0.039; CFI = 0.992.

Figure S2. Confirmatory factor analysis of the language and executive function variables at T2 (a), T3 (b) and T4 (c)
